# Successful extracorporeal membrane oxygenation for postoperative cardiopulmonary failure in newborns with congenital diaphragmatic hernia: case reports and literature reviews

**DOI:** 10.3389/fped.2023.1158885

**Published:** 2023-06-27

**Authors:** Song-Ming Hong, Xiu-Hua Chen, Si-Jia Zhou, Jun-Jie Hong, Yi-Rong Zheng, Qiang Chen, Jin-Xi Huang

**Affiliations:** Department of Cardiothoracic Surgery, Fujian Children's Hospital (Fujian Branch of Shanghai Children's Medical Center), College of Clinical Medicine for Obstetrics & Gynecology and Pediatrics, Fujian Medical University, Fuzhou, China

**Keywords:** congenital diaphragmatic hernia, cardiopulmonary failure, extracorporeal membrane oxygenation, postoperative, newborn

## Abstract

**Introduction:**

Congenital diaphragmatic hernia (CDH) is a structural defect caused by inadequate fusion of the pleuroperitoneal membrane that forms the diaphragm, allowing peritoneal viscera to protrude into the pleural cavity. Up to 30% of newborns with CDH require extracorporeal membrane oxygenation (ECMO) support. As with all interventions, the risks and benefits of ECMO must be carefully considered in these patients. Cardiopulmonary function has been shown to worsen rather than improve after surgical CDH repair. Even after a detailed perioperative assessment, sudden cardiopulmonary failure after surgery is dangerous and requires timely and effective treatments.

**Method:**

Three cases of cardiopulmonary failure after surgical CDH treatment in newborns have been reported. ECMO support was needed for these three patients and was successfully discontinued. We report our treatment experience.

**Conclusion:**

ECMO is feasible for the treatment of postoperative cardiopulmonary failure in newborns with CDH.

## Introduction

Congenital diaphragmatic hernia (CDH) is a severe congenital structural malformation in that occurs in one of every 2000–5000 newborns (1). The death rate of CDH is mainly due to severe pulmonary hypoplasia and pulmonary hypertension (PH). The main prognostic factors are preoperative pulmonary hypertension and the lung-to-head ratio (LHR) (2). Extracorporeal membrane oxygenation (ECMO) can effectively improve hypoxemia and allow patients' cardiopulmonary system to rest by drawing venous blood from the body and pumping it back into the body after oxygenation by a membrane oxygenator. With the progress in ventilator, anticoagulation, and surgical technologies, preterm birth and low birth weight are no longer contraindications for ECMO. There is little doubt that the prolonged use of ECMO is associated with increased morbidity and mortality (3). Pulmonary hypertension and acute cardiac failure after CDH surgery are important causes of death in newborns. Cardiac dysfunction resulting from physiological derangements, PPHN, or any associated congenital structural cardiac abnormality can complicate the clinical course (4). Despite careful evaluation during the preoperative and perioperative periods, there is still a possibility of catastrophic outcomes. Short-term ECMO use in the treatment of cardiopulmonary failure after CDH surgery is rarely reported. This paper reports 3 cases of sudden cardiopulmonary failure in newborns with CDH who were successfully treated by ECMO in our cardiothoracic intensive care unit and analyzes and summarizes our experience.

## Case 1

**Figure 1:**
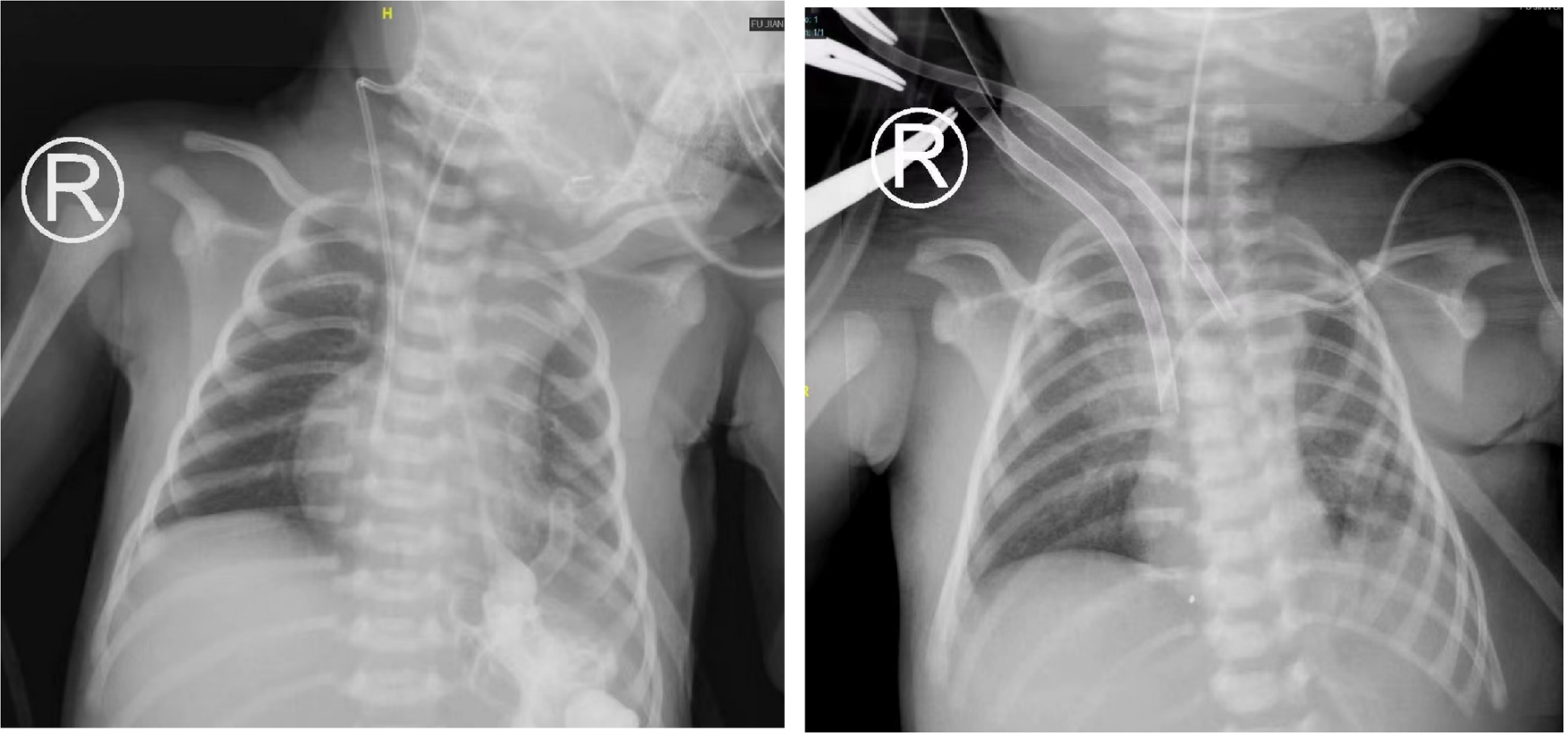
x-ray before ECMO treatment and during ECMO treatment in case 1.

A female newborn was diagnosed with CDH before delivery. The 30-week pregnancy color ultrasound showed that a left CDH and an LHR of 2.92. The baby was delivered by cesarean section at 39 ^+ 4^ weeks, with a birth weight of 2,995 g and an Apgar scores of 9–10–10. The newborn was transferred to the CICU under nasal catheter oxygen inhalation. The pulmonary artery systolic pressure estimated by transthoracic echocardiography (TTE) was approximately 57 mmHg, the patent ductus arteriosus was 7 mm in diameter with a right to left shunt, and the left ventricular ejection fraction (EF) was 65%. The patient developed shortness of breath at 6 h after birth and was treated with tracheal intubation and mechanical ventilation (HFOV: RR, 10 Hz; FiO_2_, 70%; MAP, 16 cmH_2_O; amplitude, 35 mbar). On the second day after birth, the patient underwent thoracoscopic CDH repair. After surgery, cardiac insufficiency was observed. The left ventricular EF was 31.9%, the left ventricular fractional shortening (LVFS) was 13.8%, and the pulmonary artery systolic pressure was approximately 61 mmHg. The patient received inhaled NO (INO) to reduce the pulmonary artery pressure 2 h after surgery. Positive inotropic drugs (epinephrine, norepinephrine, and dopamine) were used to improve cardiac function. At 12 h after the operation, the radial blood pressure decreased progressively, fluctuating at 40–55/20–30 mmHg. Blood gas analysis showed that a pH of 7.527, PaO_2_ of 23.5 mmHg, PaCO_2_ of 34.5 mmHg, and Lac of 18 mmol/l. The calculated oxygenation index (OI) value was 76. Echocardiographic evaluation showed a left ventricular EF of 21.9%, an LVFS of 9.22%, and a pulmonary systolic pressure of 61 mmHg. Considering the complication of left heart failure, the patient met the indications for ECMO treatment. Venoarterial (VA) ECMO was used. The right common carotid artery and internal jugular vein were incised and intubated under direct vision. The diameter of the artery cannula was 8 Fr and the depth was 3 cm. The diameter of the vein cannula was 10 Fr with a depth of 7 cm (Figure 1). The flow was 0.40 L/min (110% of the theoretical need), the centrifugal pump speed was 2,480 rpm, and the ACT was maintained at 180∼220 s. Dynamic monitoring of the brain and abdominal cavity during ECMO did not show related bleeding or thrombosis events. At 72 h after ECMO treatment, the left ventricular EF reached 55%, and the pulmonary artery pressure decreased to 43 mmHg. The patient's hemodynamics were stable 2 h after the withdrawal test. Then, ECMO was discontinued. We did not ligate the right cervical vascularity of the patient. An 8–0 Prolene was used to anastomose the vessels with a transverse suture after removing the artery and vein cannula. On the seventh day, the tracheal tube was successfully removed. The patient was discharged from the hospital on the twentieth day after the operation and followed up for six months. No surgical-related complications occurred during the follow-up period.

## Case 2

**Figure 2:**
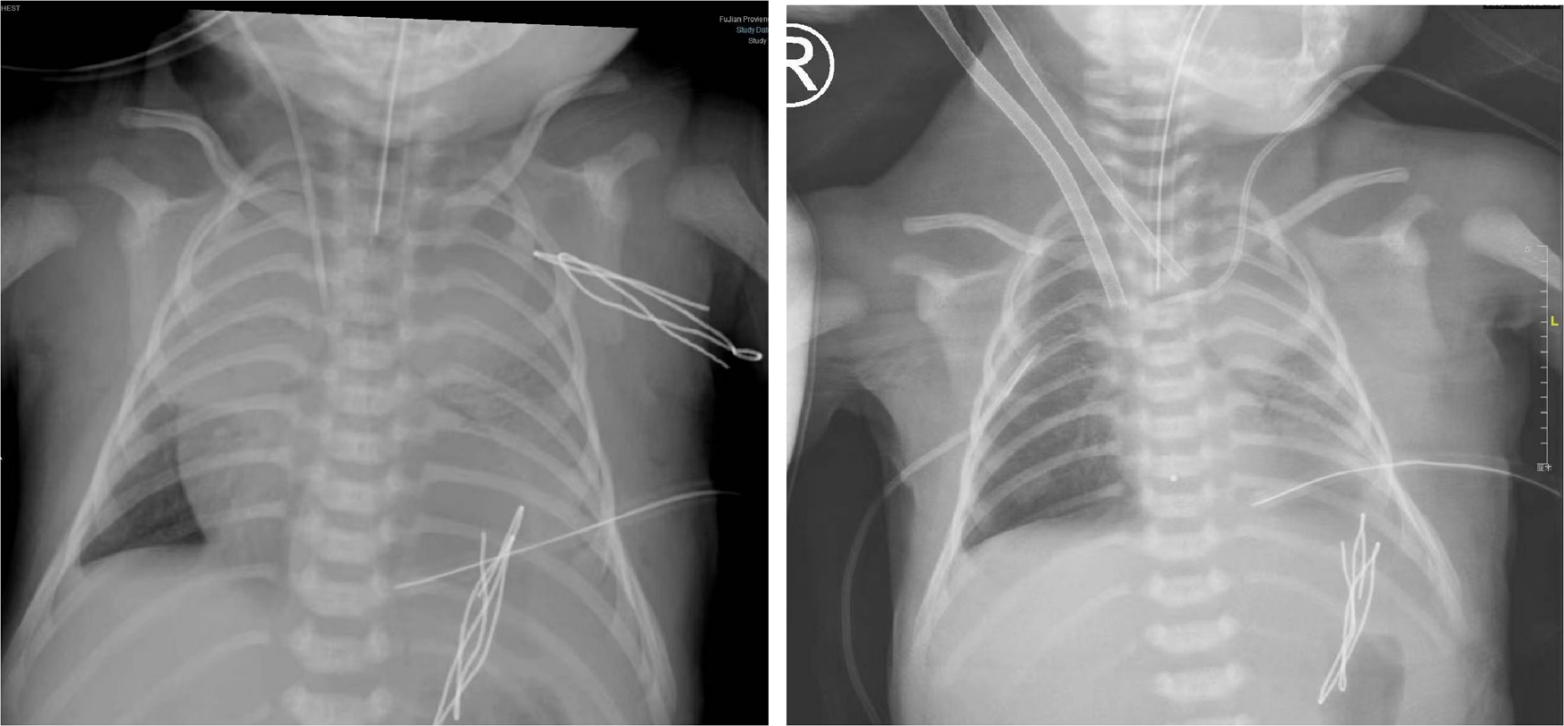
x-ray before ECMO treatment and during ECMO treatment in case 2.

A woman (G_3_P_3_) delivered a male newborn at 40 ^+ 4^ weeks of gestation, with a birth weight of 3,360 g and Apgar scores of 8–10–10. At the 31st week of pregnancy, a left CDH was diagnosed by pregnancy color ultrasound, and the estimated LHR was 1.92. Thoracoscopic CDH repair was performed on the second day after birth and the patient was in a clinically stable condition. The operation lasted 134 min. After surgery, respiratory support was given (HFOV: RR, 10 Hz; FiO_2,_ 70%; MAP, 19 cmH_2_O; amplitude, 35 mbar). Blood gas analysis was conducted two hours after surgery, showing a pH of 7.201, PaO_2_ of 56.5 mmHg, PaCO_2_ of 52.4 mmHg, and Lac of 1.8 mmol/l. On the first postoperative day, the SpO_2_ gradually decreased to 55%, the heart rate gradually decreased to 50 beats/min, and the blood pressure gradually decreased to 55/32 mmHg. Dopamine was used to improve circulation and maintain blood pressure. Blood gas analysis showed a pH of 6.942, PaO_2_ of 20.2 mmHg, PaCO_2_ of 59.8 mmHg, and Lac of 16 mmol/l. The pulmonary systolic pressure estimated by TTE was more than 100 mmHg, and the left ventricular EF was approximately 25%. The calculated OI value was 90, and the vasoactive inotropic score was 120. Then, sudden cardiac arrest occurred, which might have been due to pulmonary hypertension crisis. The duration of cardiac arrest was short because cardiopulmonary resuscitation performed for 5 min was successful. After INO treatment, the hypoxemia remained uncorrected, and cardiac arrest occurred intermittently and required cardiopulmonary resuscitation. Then, V-A ECMO was performed. The diameter of the artery cannula was 8 Fr and the depth was 3 cm. The diameter of the vein cannula was 10 Fr and the depth was 7 cm (Figure 2). The flow rate was 0.36 L/min (86% of the theoretical need). ACT was maintained at 180∼220 s. During ECMO treatment, the patient developed massive right pleural effusion. A large, yellow, leaky pleural effusion (approximately 150 ml) was drained and showed neither hemothorax nor chylothorax. On the second day of ECMO, a craniocerebral color ultrasound indicated a right temporal slightly hyperechoic area; the cerebral parenchymal echo was diffusely enhanced, and the anterior cerebral artery blood flow resistance index had decreased. The pulmonary artery pressure was 45 mmHg, and the left ventricular EF was normal, as estimated by TTE. After 72 h of ECMO treatment, we successfully discontinued ECMO, the blood pressure stabilized at 60–75/30–48 mmHg, and the heart rate stabilized at 140–150 beats/min. Unfortunately, the patient was still in a deep coma after ECMO withdrawal. No significant craniocerebral hemorrhage occurred during ECMO treatment. The brain injury caused by hypoxemia before ECMO could not be treated, and the prognosis was poor. On the seventh day after ECMO treatment, the patient's family decided to give up treatment after careful consideration.

## Case 3

**Figure 3:**
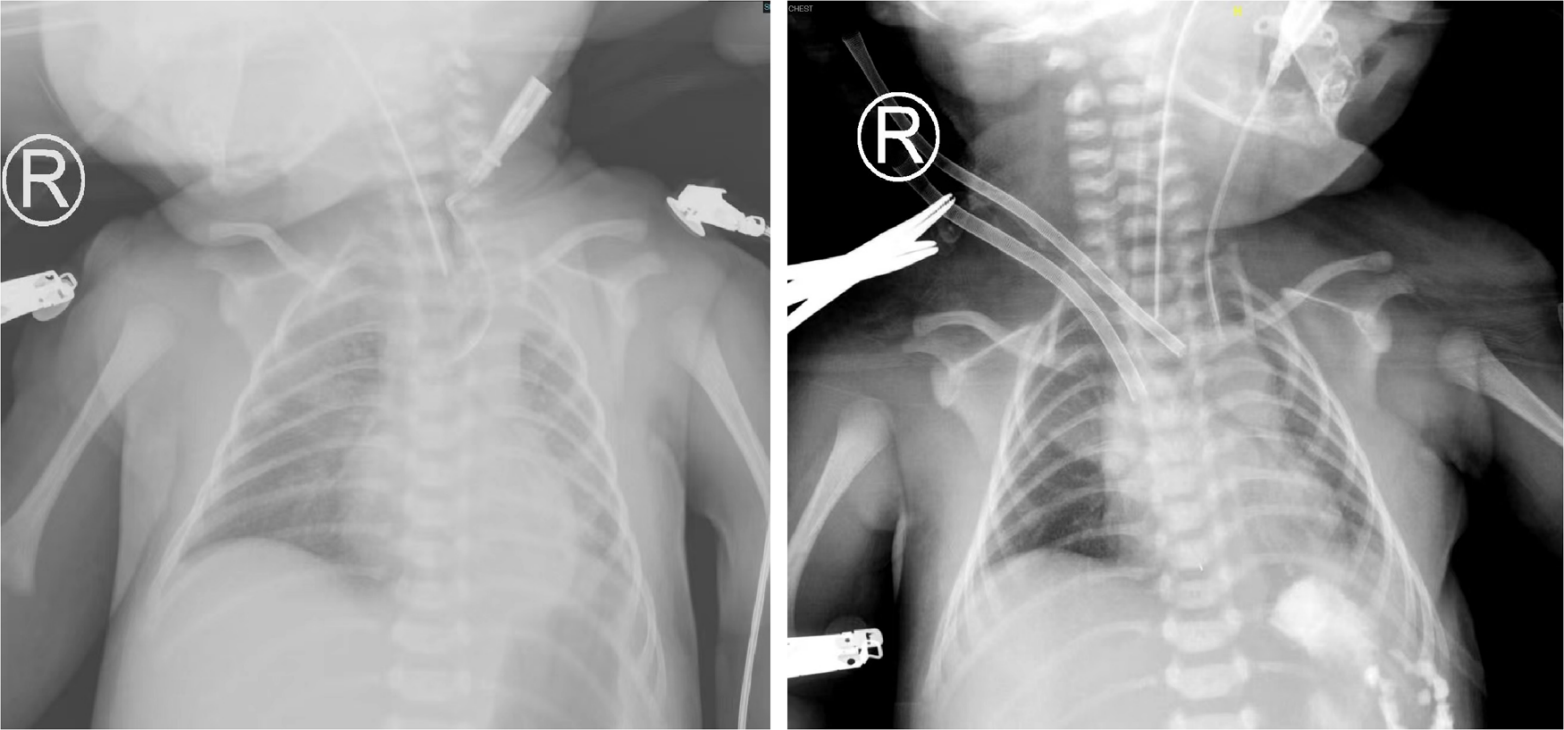
x-ray before ECMO treatment and during ECMO treatment in case 3.

A male patient was born at 38 ^+ 1^ weeks gestation by cesarean section, with a birth weight of 3,605 g and Apgar scores of 9–10–10. After birth, the patient had shortness of breath and was transferred to our hospital with mechanical ventilation support (SIMV: RR, 35 times/min; PIP, 18 cmH_2_O; PEEP, 4 cmH_2_O; Ti, 0.4 s; FiO2, 60%). Fetal color ultrasonography indicated a CDH, and the estimated LHR was 2.4. Due to the blood gas analysis suggesting respiratory acidosis, the mode was changed to HFOV (RR, 10 Hz; FiO2, 70%; MAP, 13 cmH_2_O; amplitude, 33 mbar). TTE showed that the patent ductus arteriosus was 5 mm in diameter with a right to left shunt, and the pulmonary artery pressure was 70 mmHg. The patient's blood pressure decreased 16 h after birth, fluctuating between 70 and 75/43–45 mmHg. Noninvasive hemodynamics showed that the CO was 0.4 L/min, and the CI was 2.8 L/min/m^2^. Low cardiac output was considered due to cardiac compression. Electrocardiogram indicated a first-degree atrioventricular block. Twenty-four hours after birth, the thoracic surgeon surgically repaired the diaphragm. Two hours after surgery, the patient's heart rate decreased slowly (70–85 beats/min). The arrhythmia worsened, and atrioventricular block developed, followed by intermittent ventricular fibrillation. It was not easy to restore sinus rhythm after bedside defibrillation. The patient's hemodynamics gradually became unstable. We suspected a rapid shift of the heart to the left cavity, resulting in increased arrhythmia. TTE showed that the left ventricular EF was 45% and the pulmonary artery pressure was 58 mmHg. Blood gas analysis results showed a pH of 7.423, PaO_2_ of 38.5 mmHg, PaCO_2_ of 69.2 mmHg, and Lac of 2.9 mmol/l. Considering the ECMO indication, V-A ECMO was used. The diameter of the artery cannula was 8 Fr and the depth was 3 cm. The diameter of the vein cannula was 10 Fr and the depth was 7 cm (Figure 3). The flow rate was 0.45 L/min (100 percent of the theoretical need), the centrifugal pump speed was 2,280 rpm, and ACT was maintained at 180∼200 s. The heart rate recovered to 110–120 beats/min on the first day of ECMO. At the sixtieth hour of ECMO, the left ventricle EF was 56%, the heart rate was stable at 120–130 beats/min, and no arrhythmias reoccurred. At the sixty-second hour of ECMO, ECMO treatment was stopped, and the cervical vessels were anastomosed. After ECMO treatment, TTE showed a right atrial thrombus (approximately 0.5 × 0.4 cm). Anticoagulant therapy was started immediately. On the seventh day of anticoagulant medicines, the thrombus had been completely dissolved. The patient was discharged from the hospital with daily oral aspirin anticoagulation and regular monitoring of coagulation function.

## Discussion

CDH is a morphological disease caused by an abdominal visceral hernia in the pleural cavity due to incomplete fusion of the diaphragm. Lung hypoplasia and abnormal development of the pulmonary vasculature with hyperreactivity lead to persistent pulmonary hypertension in newborns (PPHN) (5). Episodes of hypoxia and hypercapnia can exacerbate PPHN. This vicious positive cycle can lead to severe morbidity and mortality. ECMO can be used in infants with CDH whose clinical status deteriorates, such as those who experience perioperative secondary pulmonary hypertension crisis and cardiac failure (6). The three patients reported in this study received ECMO treatment for cardiopulmonary failure after CDH surgery.

To improve the treatment of CDH, it is essential to manage PPHN and choose the optimal time for diaphragm repair (7). In fact, pulmonary hypertension can be worsened by a variety of factors, such as severe CDH, unstable circulation, and acidosis, with surgery as the main reason (8–10). Some scholars have proposed strategies to reduce acidosis during surgery, such as reducing the pressure of artificial pneumothorax after returning the herniated viscera to the abdomen and significantly improving hypercapnia and acidosis (11, 12). However, up to 30% of CDH patients are still unstable after surgery, and some patients with severe cardiac dysfunction need ECMO treatment (13, 14). In Case 2, the patient underwent thoracoscopic surgery for more than 2 h, which might have been the primary reason for the pulmonary hypertension crisis. Severe hypoxemia and the pulmonary artery pressure that gradually increased to 100 mmHg might have led to the difficulty in cardiopulmonary resuscitation. The increasing pulmonary artery pressure caused by this procedure was observed to be reversible and decreased to 45 mmHg after three days of ECMO treatment. Although cardiac function recovered after ECMO treatment, difficulties in resuscitation caused by craniocerebral injury resulted in a poor prognosis. The real-time monitoring of the pulmonary artery pressure in newborns with CDH after surgery is essential. A gradually increasing pulmonary artery pressure may be an important signal for ECMO intervention.

Many studies have reported the importance of TTE in patients with CDH. Early cardiac insufficiency and severe pulmonary hypertension are independent predictors of adverse outcomes in “low-risk” patients with CDH (15–17). A Japanese study showed that the EF of patients with a left CDH decreased to varying degrees after surgery, even lower than 30% (18). Siebert et al. found that neonates with severe CDH often had left ventricular hypoplasia (19). Altit et al. studied changes in echocardiography in infants with CDH and severe PH and found that impaired cardiac function was a better predictor of the need for ECMO than the severity of PH (20). Postoperative low left ventricular output was observed early in Case 1. Despite early relief of pressure on the heart, patients with left CDH appear to have more cardiac insufficiency after surgical repair. The reason might be that left ventricular septum deviation affects left ventricular output function due to the postoperative right ventricular dysfunction caused by pulmonary artery pressure aggravation. Considering that the increased pulmonary artery pressure was treated by HFOV combined with INO, left heart function continued to decline. The possible reason for this is that tensile diaphragm repair placed more mechanical stress on the left heart during cardiac displacement, which mainly manifested as reduced systolic and diastolic function. In this case, left ventricular function significantly improved on the third day of ECMO. We suggest that short-term ECMO treatment for cardiac failure played an important supporting role in these patients. Frequent postoperative TTE was essential for the dynamic assessment of left heart function, even though the LHR assessed during pregnancy indicated low risk.

Arrhythmia is rarely reported after CDH surgery, and the relationship between diaphragmatic hernia and atrioventricular conduction disorder remains unclear. Elevated intrathoracic pressure (such as pneumothorax) associated with atrioventricular conduction disorders has occurred in only a few patients (21). In Case 3, there was no other reason for atrioventricular block, which indicated that CDH surgery might have been the reason for postoperative arrhythmia aggravation. After ECMO support, the arrhythmia gradually resolved. Cardiac dysfunction caused by electrophysiological disorders, PPHN, or any related congenital cardiac abnormality could complicate clinical processes and require ECMO support (22). ECMO has been used as hemodynamic protection for aggressive antiarrhythmic medical treatment in pediatric patients, with a survival rate >80%. It could be recommended as a rescue and support therapy in pediatric patients requiring aggressive antiarrhythmic medical treatment (23). Arrhythmias requiring ECMO support are rare in infants without structural congenital heart disease. Given the favorable survival rate, earlier and more aggressive ECMO use may lead to improved outcomes (24). Patients with postoperative severe arrhythmias might benefit from short-term treatment with ECMO in an emergency.

Indications for ECMO initiation in infants with CDH are inconsistent. Many factors may affect all patients, making it difficult to decide when to start ECMO treatment. Relative indications for ECMO have been studied by multiple investigators and include an elevated OI, persistently low oxygen saturation despite maximal ventilator assistance, hypercarbia, and an elevated alveolar-arterial oxygen gradient (A-aDO_2_) (25, 26). All of our cases were consistent with such indications. V-A ECMO may have some advantages for newborns with cardiac dysfunction, allowing the heart to rest and maintaining systemic sound output (27–29). V-A ECMO was selected for all our patients. Cardiopulmonary function recovered satisfactorily on the third day of ECMO treatment. No acute kidney injury or significant ECMO-related intracranial hemorrhage occurred during treatment. Therefore, based on our experience, we recommend using V-A ECMO to treat postoperative cardiopulmonary failure in newborns with CDH.

## Conclusion

ECMO is feasible for the treatment of postoperative cardiopulmonary failure in newborns with CDH. Timely implementation is of positive significance for improving the survival rate of newborns. However, only three cases were reported in this paper, and more patients need to be evaluated to obtain more experience in ECMO treatment after CDH surgery.

## Data Availability

The raw data supporting the conclusions of this article will be made available by the authors, without undue reservation.
